# Effects of long term anti epileptic drugs on serum vitamin D levels and bone profile in a cohort of Sri Lankan children

**DOI:** 10.1186/1687-9856-2015-S1-P66

**Published:** 2015-04-28

**Authors:** N Ginige, K S H de Silva, J K Wanigasinghe, N S Gunawardane, T M J Munasinghe

**Affiliations:** 1 Lady Ridgeway Children’s Hospital, Colombo, Sri Lanka; 2 Department of Paediatrics, Faculty of Medicine, Colombo, Sri Lanka; 3 Department of Community Medicine, Faculty of Medicine, Colombo, Sri Lanka; 4 Medical Research Institute, Colombo, Sri Lanka

## Aims

To demonstrate association between Vitamin D levels and AED usage.

To demonstrate effects of long term antiepileptic drug use on bone metabolism –eg: Serum alkaline phosphatase (ALP), calcium.

## Method

A retrospective cohort study was performed on 205 children aged 1−12 years presented to a tertiary care hospital in Sri Lanka;119 with epilepsy, exposed to AEDs more than 2 years and 86 who are unexposed to AED. Vitamin D levels and bone profile were analyzed by chemiluminescent auto analyzer and photometric methods respectively. The prevalence of Vitamin D deficiency (< 20ng/ml) among children exposed and unexposed to AED was compared. Among exposed the effect of AED combinations on Vitamin D deficiency were assessed. Similarly bone profile was compared in the two groups.

## Results

A higher proportion of children on AED were deficient in Vitamin D (53.7%) compared to children who are not on AED (45.3%) (p=0.233).

Difference of Vitamin D deficiency among those on poly AED therapy (59.5%) and single AED (50.6%) were non-significant (p=0.353). Similarly, differences of Vitamin D deficiency in those on Carbamezapine mono therapy (50% n=18/36) and Sodium Valproate mono therapy (60.7%; n=17/27) were non-significant (p=0.4).

The mean ALP among children on AED was significantly higher (599.4 ±178.96) compared to children not on AED (420.6±152.59)(p=0.044).The mean serum calcium among children exposed to AED (2.39+0.15)and those not exposed to AED (2.36+0.17) were similar(p=0.34).

## Conclusions

Vitamin D deficiency was not significantly associated with long term AED use in children with epilepsy. Being on polytherapy over monotherapy or being on carbamezapin over sodium Valproate was not associated with Vitamin D deficiency.

However a significantly higher than normal levels of ALP was associated with long term use of AED which needs further evaluation.

